# Clouston Syndrome: 25-year follow-up of a patient*

**DOI:** 10.1590/abd1806-4841.20153990

**Published:** 2015

**Authors:** Lívia Arroyo Trídico, João Roberto Antonio, Eurides Maria de Oliveira Pozetti, Ana Maria Mendes Rosa, Carlos Roberto Antonio

**Affiliations:** 1 Faculdade de Medicina de São José do Rio Preto (FAMERP) - São José do Rio Preto (SP), Brazil

**Keywords:** Ectodermal dysplasia, Hypotrichosis, Keratoderma, palmoplantar

## Abstract

Clouston syndrome is a rare genodermatosis that affects skin and annexes. It is a form of ectodermal dysplasia characterized by generalized hypotrichosis, palmoplantar hyperkeratosis and nail dystrophy. This paper reports a 25-year follow-up of a patient with Clouston syndrome, from childhood to adulthood, monitoring diagnosis and clinical course of the disease.

## INTRODUCTION

Clouston syndrome or hidrotic ectodermal dysplasia is a rare genetic disease which involves the skin and annexes.^1^ It is a form of ectodermal dysplasia initially described in 1895 and later reported in Canadian families by Clouston in 1939.^2^

Ectodermal dysplasia describes a group of genetic diseases characterized by dysplasia of tissues that originate from the ectoderm and, occasionally, of tissues that originate from the mesoderm during embryonic development.^3^ It occurs approximately in 1 in every 100,000 live births.^4^ This disorder is divided into two main groups: hypohidrotic and hidrotic.

Hypohidrotic ectodermal dysplasia is the most common (80% of cases) and is characterized by absence or reduced number of sweat glands, associated with hypodontia, hypotrichosis and facial dimorphism.^4^ On the other hand, in hidrotic ectodermal dysplasia there is no alteration in sweat glands and dentition is normal, but hair and nails are affected.^5^

Hidrotic ectodermal dysplasia or Clouston syndrome is characterized by the main triad: nail dystrophy, generalized hypotrichosis and palmoplantar hyperkeratosis.^1^ The affected patients present scarce hair and nail dystrophy, both noticeable since the first months of life. During infancy, hair is fragile and thin and its progressive loss may lead to total alopecia in puberty. Nails are whitish during childhood and gradually become dystrophic, thin and distally separated from the nail bed. Nail clubbing may occur. Palmoplantar keratoderma may develop in childhood and progress with age. Clinical characteristics vary greatly among individuals, even within the same family.^6^

In addition, some patients may present skin hyperpigmentation, more evident on the joints.^5,7^ Strabismus, conjunctivitis, cataracts, deafness, polydactyly and syndactyly may occur.^7^ Eccrine syringofibroadenomas have been reported in some patients.^8^ Facial dimorphism is not present.^9^

Clouston syndrome has a dominant autosomal pattern, therefore most patients have an affected family member, although new mutations have been reported as well.^6^ Changes in gene *GJB6*, located in chromosome 13 (locus 13q11-q12) are responsible for the syndrome, since the gene is involved in the differentiation and growth of keratinocytes.^9^ This gene codifies the cell-junction protein, connexin 30, a transmembrane protein which facilitates intercellular communication and is present in the stratum corneum, sweat glands and hair follicles. Connexins have an essential role in the control of cellular growth and development, besides responding to several stimuli.^5,9^

## CASE REPORT

Female patient, 25 years old, from the state of São Paulo.

When she was ten months old, her mother who brought her to our clinic reporting that the child had had absence of hair, eyelashes and eyebrows since birth. An anatomopathological examination performed on the scalp revealed alopecia. One year later she presented desquamative lesions on her fingertips and dystrophic fingernails. At two years and six months of age, hyperkeratotic and desquamative plaques appeared on her palms.

**Figure 1:**
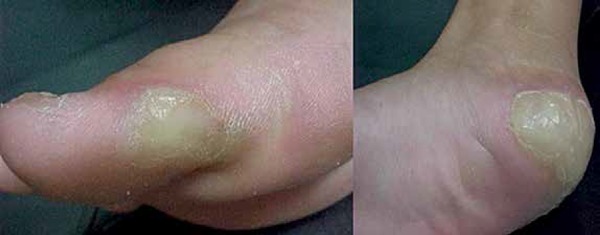
Plantar Hyperkeratosis

**Figure 2:**
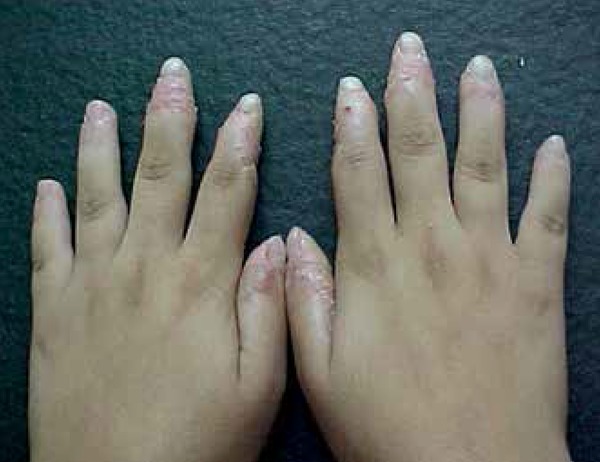
Distal tapering of fingers

At eight years of age the patient presented a clinical picture of total alopecia and nail dystrophy. Hyperkeratotic plaques, initially present on her palms, involved the soles of feet as well (Figure 1). Besides, she presented a distal tapering of fingers, which at radiological examination showed reduction of soft tissues in distal extremities of fingers, acquiring a triangular configuration, and bone structures of normal appearance (Figure 2). A biopsy was done which revealed absence of hair follicles, but sweat glands with normal features. The patient did not present changes in sudoresis, dentition or hearing.

When she was 14 years old, after being evaluated by a psychologist, emotional damage was observed and depression secondary to the pathology was diagnosed. Moreover, learning difficulties were noticed and light mental retardation diagnosed. At the time, the patient wore a hairpiece and presented remission of palmoplantar keratoderma after the introduction of topical keratolytics.

**Figure 3:**
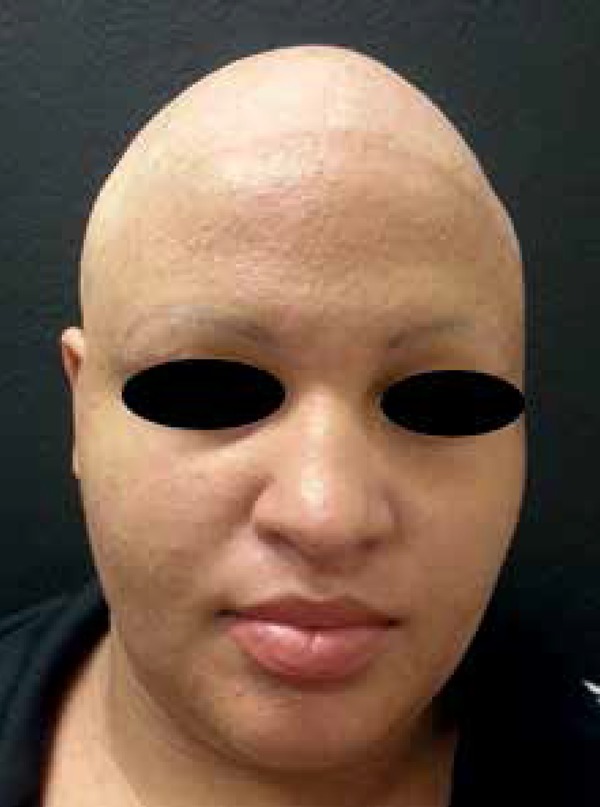
The patient at 25 years of age

Patient treatment continued with multidisciplinary follow-up (dermatology, psychology and genetics). The same patient has two children which were not affected by the syndrome and are healthy. The patient's parents are phenotypically normal with no consanguinity. Genetic study did not reveal similar cases in the family. Currently at 25 years of age, the patient is periodically monitored and deals with her pathology in a very conscious way, continuing with the treatment for palmoplantar keratoderma (Figure 3).

## DISCUSSION

Clouston syndrome affects more than one individual of the same family, since it is a dominant autosomal genetic disease.^6^ In the present case, which is sporadic in the family, we face a new mutation. Baris et al., 2008, described a new mutation in gene *GJB6* that affected mother and son in the same family, with no affected relative on the mother's side. Smith, Morley and McLean, 2002, also described a new mutation.^10^ The proportion of new mutation cases is unknown, but it is presumed to be very small.^6^

Because it is a rare syndrome, follow-up reports of patients diagnosed with Clouston syndrome do not exist. By means of this case, we aim to report the 25-year follow-up of a patient, from childhood to adult life. It was possible to observe signs of the disease present since birth that intensified as the patient grew, adding important clinical data for diagnostic clarification.

We could also observe the obstacles faced by the patient at different phases of life. In her infancy, her mother worried searching for a concrete diagnosis. During adolescence, a depressive episode due to insecurity and low self-esteem. In her adult life, pregnancy and risk of genetic transmission to offspring. The patient, whose husband was not affected, was informed about the 50% risk of recurrence in her offspring, but she decided to have two children anyway.

Early diagnosis of this disease has great value, because the patients are affected functionally, psychologically and socially. Multidisciplinary follow-up is paramount to ensure information to the patient, control of treatable diseases, emotional support and genetic counseling.
